# Characterizing advanced Parkinson’s disease: OBSERVE-PD observational study results of 2615 patients

**DOI:** 10.1186/s12883-019-1276-8

**Published:** 2019-04-02

**Authors:** Alfonso Fasano, Victor S. C. Fung, Leonardo Lopiano, Bulent Elibol, Irina G. Smolentseva, Klaus Seppi, Annamária Takáts, Koray Onuk, Juan Carlos Parra, Lars Bergmann, Kavita Sail, Yash Jalundhwala, Zvezdan Pirtosek

**Affiliations:** 1 Edmond J. Safra Program in Parkinson’s Disease and Morton and Gloria Shulman Movement Disorders Clinic, Toronto Western Hospital and Division of Neurology, UHN, Division of Neurology, University of Toronto, 399 Bathurst St, 7McL412, Toronto, ON M5T 2S8 Canada; 2 Krembil Research Institute, 399 Bathurst St, Toronto, Ontario M5T 2S8 Canada; 3 Movement Disorders Unit, Department of Neurology, Westmead Hospital, Cnr Hawkesbury Road and Darcy Road, Westmead, NSW 2145 Australia; 4 Sydney Medical School, University of Sydney, Edward Ford Building A27, Sydney, Australia; 5 Department of Neuroscience, University of Turin, Via Cherasco 15, 10126 Turin, Italy; 6 Department of Neurology, Hacettepe University Hospitals, Hacettepe Mh, 06239 Ankara, Turkey; 7 Department of Neurology, Russian Postgraduate Medical Academy, 2-second Botkinsky travel, 5, 125284 Moscow, Russia; 8 Department of Neurology, Medical University Innsbruck, Innrain 52, Christoph-Probst-Platz, 6020 Innsbruck, Austria; 9 Neurological Clinic of Semmelweis University, Balassa J.u.6, Budapest, H-1083 Hungary; 10 AbbVie, Inc, 1400 Sheridan Road, North Chicago, IL 60064 USA; 11 Department of Neurology, University Medical Center Ljubljana, Zaloška cesta 2, 1000 Ljubljana, Slovenia

**Keywords:** Advanced Parkinson’s disease, Device-aided treatment, Quality of life

## Abstract

**Background:**

There are currently no standard diagnostic criteria for characterizing advanced Parkinson’s disease (APD) in clinical practice, a critical component in determining ongoing clinical care and therapeutic strategies, including transitioning to device-aided treatment. The goal of this analysis was to determine the proportion of APD vs. non-advanced PD (non-APD) patients attending specialist PD clinics and to demonstrate the clinical burden of APD.

**Methods:**

OBSERVE-PD, a cross-sectional, international, observational study, was conducted with 2615 PD patients at 128 movement disorder centers in 18 countries. Motor and non-motor symptoms, activities of daily living, and quality-of-life end points were assessed. The correlation between physician’s global assessment of advanced PD and the advanced PD criteria from a consensus of an international group of experts (Delphi criteria for APD) were evaluated.

**Results:**

According to physician’s judgment, 51% of patients were considered to have APD. There was a moderate correlation between physician’s judgment and Delphi criteria for APD (K = 0.430; 95% CI 0.406–0.473). Activities of daily living, motor symptom severity, dyskinesia duration/disability, “Off” time duration, non-motor symptoms, and quality-of-life scores were worse among APD vs. non-APD patients (*p* < 0.0001 for all). APD patients (assessed by physicians) had higher disease burden by motor and non-motor symptoms compared with non-APD patients and a negative impact on activities of daily living and quality of life.

**Conclusions:**

These findings aid in identifying standard APD classification parameters for use in practicing physicians. Improvements in identification of APD patients may be particularly relevant for optimizing treatment strategies including transitioning to device-aided treatment.

**Electronic supplementary material:**

The online version of this article (10.1186/s12883-019-1276-8) contains supplementary material, which is available to authorized users.

## Background

Patient progression to advanced Parkinson’s disease (APD) has conventionally not been well-defined. The worsening of cardinal features of PD (e.g. tremor, rigidity, akinesia, and postural instability) are inevitable as disease progression occurs. The onset of motor fluctuations and/or levodopa-induced dyskinesia is commonly accepted as a marker of progression of the disease; however, determining the point at which a patient is classified as having APD is not distinct and imposes continued challenges, particularly when determining appropriate therapeutic recommendations for optimization of patient outcomes. Although commonly used in routine clinical practice to identify the clinical stages of PD, the Hoehn and Yahr scale has limitations [1], including limited capture of motor and non-motor symptoms (NMS) and inability to measure quality of life (QoL) [2–4].

As PD symptoms become increasingly refractory to oral treatments, device-aided treatment (DAT) (i.e. treatment that extends beyond oral medications) such as stereotactic surgery for deep brain stimulation (DBS) and infusion therapies—namely continuous subcutaneous apomorphine infusion (CSAI) or levodopa-carbidopa intestinal gel (LCIG)—may be considered [5, 6]. Switching from oral PD treatment to invasive therapeutics is typically determined in movement disorder centers/clinics that provide comprehensive treatment by a multidisciplinary expert group. Even within these clinics there are variable reports on percentages of APD patients [7–9], highlighting potential discrepancies between identification, categorization, and total number of APD patients. Differences in practice patterns between general neurologists and movement disorder specialists may contribute to these discrepancies. Consistent identification and categorization of APD patients in movement disorder clinics is critical for these centers to provide sufficient resources for patient education for APD symptom management and key information on DAT. Standardized criteria may also have an educational effect for the non-specialized neurologist who can then monitor patients for specific symptoms and refer patients in real time when treatment optimization is still possible.

The OBSERVE-PD (OBSERVational, cross-sEctional PD) study was designed to determine the proportion of patients with APD in movement disorder clinics in different regions. Predefined objectives included the characterization of clinical and non-clinical features based on physician’s judgment and newly developed Delphi criteria for APD, as well as a comparison of the two assessments’ determination of APD [10]. Additionally, characteristics of APD patients were examined based on DAT-eligibility.

## Methods

### Study setting and patient selection

Movement disorder clinics were selected based on DAT availability (i.e. LCIG, CSAI, or DBS) offered by an expert/specialist team. This observation study included PD patients who were attending a routine clinical visit or were inpatients at participating movement disorder clinics. Consecutive patients were asked for their interest in participation and were enrolled in the study at each site to avoid selection bias. To be included in the study, patients had to be at least 18 years old with a clinical diagnosis of PD. Patients had to sign an authorization form to disclose personal health information and an informed consent form and be fluent in the language of the provided patient questionnaire. Patients were excluded if in the “Off” stage at the time of visit, if there was significant uncertainty around the PD diagnosis (i.e. symptoms suggesting non-idiopathic PD such as early falls or early autonomic disturbances, lack of levodopa responsiveness, supranuclear gaze disturbances, history of repeated strokes with stepwise progression of parkinsonism), or if patients were participating in a concurrent clinical study. The study was approved by local ethics committees and performed according to the International Conference on Harmonization and Good Clinical Practice requirements, in accord with the principles of the Declaration of Helsinki.

### Study assessments

Following signed consent, data were collected from each patient at a single study visit. Patient records provided demographic information (age, sex, race, patient residence, and caregiver support), cognitive function (assessed in the case report form in a general manner, not using a scale), PD-related information (date of diagnosis, referral history, and disease stage based on physician’s judgment), PD treatment information (PD treatment history, patient qualification/eligibility for invasive therapy according to physician’s judgment, current PD treatment [s] and physician’s assessment of response to current treatment), and comorbidities. Patient qualification/eligibility for DAT was not mandated by certain eligibility criteria set forth by the study and was instead dictated by the movement disorder specialist’s judgment for potential patient candidacy for DAT. Motor symptoms assessed during the study visit included Unified Parkinson’s Disease Rating Scale (UPDRS) in “On” stage (includes Part II [activities of daily living, ADL], Part III [motor complications], and Part IV [items 32, 33, 34, and 39; complications of therapy]), and UPDRS Part V (modified Hoehn and Yahr scale staging). Non-motor symptoms were assessed using the Non-motor Symptom Scale (NMSS), and QoL was assessed using the 8-item Parkinson’s Disease Quality of Life Questionnaire (PDQ-8).

### Delphi criteria for APD

Delphi criteria for APD were developed as a staging tool to help clinicians identify patients with APD using consistent characterization, which therefore will assist in optimizing patient treatment care (e.g. DAT eligibility) [10]. The Delphi versus physician judgment comparison was a pre-defined end point. Patients were first assessed by the investigator using their own physician’s judgment and then assessed using the Delphi criteria. Independent assessments were not possible as the outcomes were determined by the same investigator. In this study, patients were assessed by movement disorder specialists using 11 indicators (Table 1) of suspected APD identified through a Delphi consensus methodology by an international movement disorder specialist’s expert group. Patients having any of the indicators were identified as having APD [10]. As APD was determined by individual physician judgment, no comparison to other scales was conducted. Patients with ongoing DAT were not assessed using the Delphi criteria as the comparison was considered invalid as they would usually experience better clinical outcomes.

**Table 1:** Delphi criteria for APD [10]

Delphi consensus criteria questions	Patient has
1. Troublesome motor fluctuations, severity level	Moderate or severe
2. “Off” time, hours/waking day	≥ 2 or < 2
3. Nighttime sleep disturbances, severity level	Moderate or severe
4. Troublesome dyskinesia, hours/waking day	2–3 or > 3
5. Non-motor fluctuations present	Yes
6. “Off” time at least every 3 h	Yes
7. ≥ 5 times daily oral levodopa dosing	Yes
8. Activities of daily living limitation, severity level	Moderate or severe
9. Falling, frequency	Most of the time or all the time
10. Dementia, severity level	Moderate or severe
11. Psychosis, severity level	Moderate or Severe

### Safety

This observational study was not designed to identify or quantify safety aspects of any therapy. Any product-related events were reported in accord with local laws and regulations to the relevant regulatory authority and/or drug marketing authorization holder. Patient data were documented on data report forms. Representatives at each center were asked to complete a site information form.

### Statistical analysis

Statistical analyses were performed using the SAS® package, version 9.2 (SAS Institute, Cary, NC). Data were summarized using descriptive statistics. The primary end point was the proportion of PD patients who had APD according to physician judgment. A secondary end point compared physicians’ judgment with the Delphi criteria for APD. A multivariable logistic regression analysis was applied with physicians’ assessments of APD as target variable and 32 potential prognostic factors including all Delphi criteria for APD, UPDRS II to V, UPDRS IV item 32, UPDRS IV item 33, UPDRS IV item 34, UPDRS item 39, NMSS score, PDQ-8, time since diagnosis, current invasive PD treatment, gender, race, education, motor fluctuations, caregiver support, comorbidities, geographic region, occupation, and type of residence. The correlation between the Delphi criteria for APD and physicians’ assessments of APD were confirmed with an additional sensitivity analysis by excluding ongoing DAT patients to eliminate any potential masking effect of the invasive treatment. Two-sided 95% CIs were provided for comparative end points; CIs and *p* values (two-sample *t* test) were calculated for differences between APD and non-advanced PD (non-APD) patients.

A post hoc analysis assessed baseline demographics and disease characteristics of APD patients who were assessed as eligible or ineligible for DAT by the treating physician. Patient characteristics were analyzed using descriptive statistics. An analysis of variance compared disease characteristics of patients eligible for DAT (ongoing DAT versus planned DAT versus no DAT) and patients ineligible for DAT with *t* tests for pairwise comparisons between each group for selected scale scores.

## Results

### Comparisons between APD and non-APD patients

This study evaluated 2615 PD patients from 128 movement disorder clinics in 18 countries. According to physician’s assessments, 51.3% (*n* = 1342) of recruited PD patients had APD; this proportion varied regionally (range: 24 to 82%; Table 2). There were no differences between APD and non-APD patient percentages regarding age, sex, and living situation (Table 3). Notable differences were observed in caregiver support status, which was needed by most APD patients (69.1%), whereas only 25.8% of non-APD patients had caregiver support. Most patients (APD and non-APD) were on oral levodopa/carbidopa or oral dopamine agonists at the time of the study (Additional file 1: Table S1).

**Table 2:** APD and non-APD by country based on investigator’s judgment

Country, n (%)	APD	Non-APD
Australia	61 (61)	39 (39)
Austria	73 (61)	47 (39)
Belgium	77 (46)	90 (54)
Canada	78 (32)	164 (68)
Croatia	32 (58)	23 (42)
Czech Republic	60 (82)	13 (18)
Germany	122 (69)	55 (31)
Greece	65 (38)	105 (62)
Hungary	50 (50)	50 (50)
Ireland	29 (24)	90 (76)
Israel	70 (58)	50 (42)
Italy	60 (43)	80 (57)
Romania	95 (59)	66 (41)
Russia	106 (42)	144 (58)
Slovakia	102 (80)	26 (20)
Slovenia	45 (46)	54 (54)
Switzerland	93 (69)	41 (31)
Turkey	124 (48)	136 (52)

*ADP* advanced Parkinson’s disease, *non-APD* non-advanced Parkinson’s disease

**Table 3:** Characteristics of ADP and non-ADP based on physician’s judgment

	APD, *N* = 1342		Non-APD, *N* = 1273	
Characteristics	n/N (%)	Mean (SD)	n/N (%)	Mean (SD)
Age, years		67.6 (9.4) *n* = 1338		66.4 (10.3) *n* = 1272
Sex, male	817/1342 (61)		734/1273 (58)	
Living at home	1304/1342 (97)		1264/1273 (99)	
Required caregiver support, yes	917/1327 (69)		328/1270 (26)	
Time since diagnosis, years		11.0 (5.8) *n* = 1304		4.3 (3.7) *n* = 1249
Motor fluctuations present, yes	1167/1342 (87)		295/1273 (23)	
Duration of motor fluctuations, years		4.9 (3.9) *n* = 1151		2.3 (2.1) *n* = 272
UPDRS V: Modified Hoehn & Yahr score		2.9 (0.8)^a^ *N* = 1342		2.0 (0.6)^a^ *n* = 1272
Eligible for invasive treatment options, yes	882/1342 (66)		127/1272 (10)	
Status of invasive treatment for eligible patients				
Ongoing	384/882 (44)		15/127 (12)	
Decided at visit to start	164/882 (19)		17/127 (13)	
No	332/882 (38)		91/127 (72)	
Missing	2/882 (0.2)		4/127 (3.1)	

^a^The UPDRS was measured in the “On” state. Statistical significance based on a *t* test comparison between APD and non-APD patients are indicated at the *p* < 0.0001. All proportions except for the invasive treatment status included patients with no missing data. *APD* advanced Parkinson’s disease, *non-APD* non-advanced Parkinson’s disease, *PD* Parkinson’s disease, *SD* standard deviation, *UPDRS* Unified Parkinson’s Disease Rating Scale

**Fig. 1:**
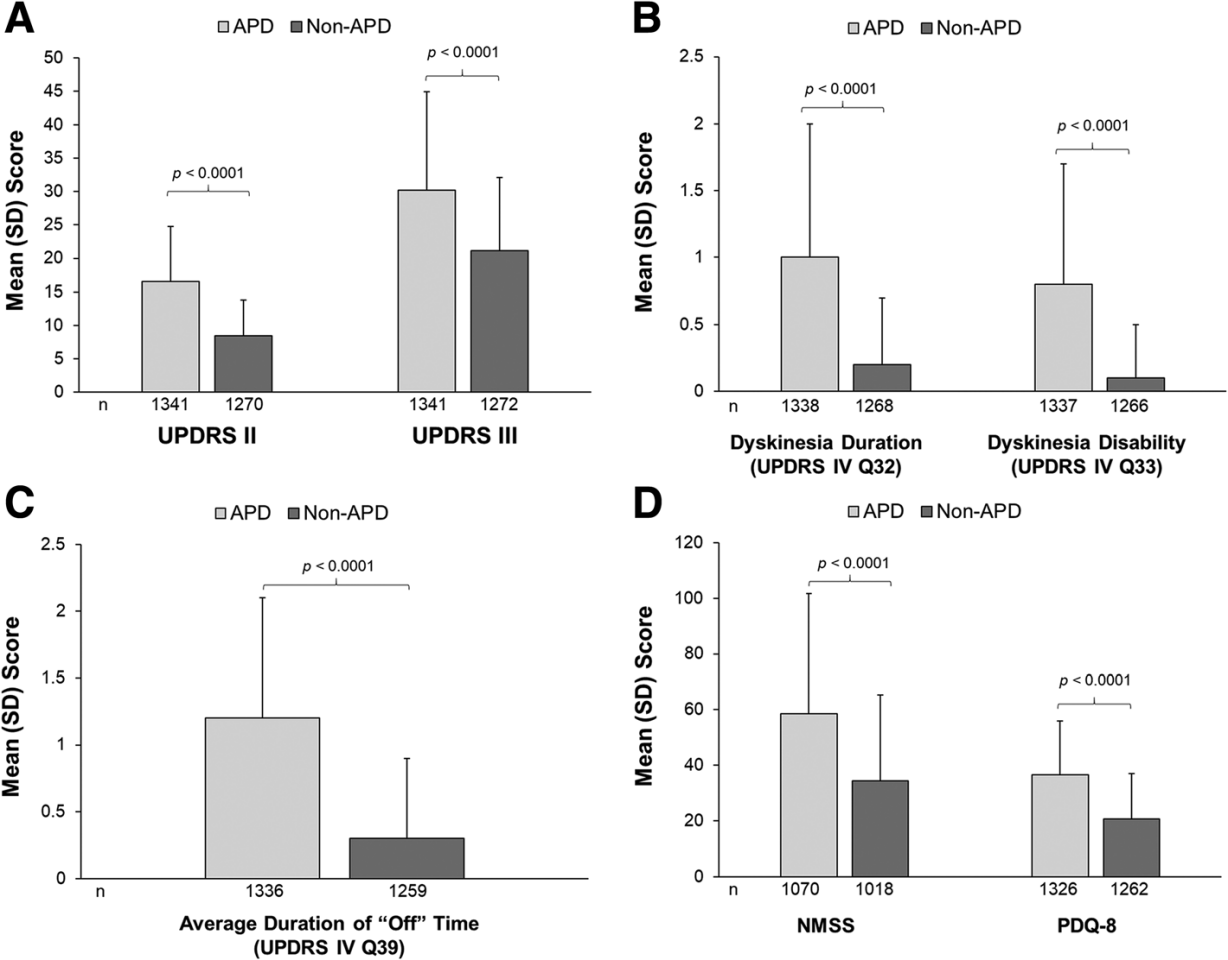
Clinical PD assessments in APD versus non-APD patients. **a** Activities of daily living (UPDRS II) and motor symptom severity (UPDRS III), **b** dyskinesia duration and disability (UPDRS Part IV Q32 and Q33), **c** average duration of “Off” time (UPDRS Part IV Q39), and (**d**) NMS burden and quality of life (PDQ-8). Error bars indicate the standard deviation. *p* values from a paired *t* test indicate statistical significance. APD = Advanced Parkinson’s disease; non-APD = Non-advanced Parkinson’s disease; NMSS = Non-motor Symptom Scale; PDQ-8 = Parkinson’s disease 8-item questionnaire; SD = Standard deviation; UPDRS = Unified Parkinson’s Disease Rating Scale

Patients with physician-assessed APD had a greater disease burden than non-APD (Fig. 1). Mean scores for ADL, motor symptom severity, dyskinesia duration and disability, average duration of “Off” time, NMS, and QoL were all significantly worse among APD patients than non-APD patients (*p* < 0.0001 for all). Most (87%) APD patients experienced motor fluctuations (94% of DAT-eligible patients and 74% of DAT-ineligible patients).

### Delphi criteria for APD

There was a moderate correlation between physician’s judgment of APD and classification based on all indicators of APD identified by the Delphi criteria (K = 0.441; 95% CI, 0.408–0.473); there was also a moderate correlation between individual items from the Delphi criteria for APD and physician’s judgment (Table 4). A sensitivity analysis, which excluded patients with ongoing DAT, confirmed the moderate correlation (K = 0.430; 95% CI, 0.397–0.464; Additional file 1: Table S2). Correlations with physician’s judgment were significant for the Delphi criteria of ≥5 times daily oral levodopa dosing (*p* < 0.0001) and level of limitation of activities of daily living (*p* = 0.0022). Physician’s judgment of APD also correlated with UPDRS II score (*p* < 0.05)**,** Hoehn and Yahr score (*p* < 0.0001), NMSS score (*p* < 0.05), time since diagnosis (*p* < 0.0001), current invasive PD treatment (*p* < 0.0001), motor fluctuations (*p* < 0.0001), existence of caregiver support (*p* < 0.001), and type of residence (*p* < 0.05) (Additional file 1: Table S3).

**Table 4:** Correlations between physician’s judgment of APD and individual Delphi criteria for APD

		Odds ratio			
Prognostic parameter	Regression coefficient	Adjusted estimate	95% Wald CI		*p* value
Troublesome motor fluctuations (severity level, moderate/severe versus mild)	0.1122	1.119	0.743	1.684	0.5909
“Off” time (hours/waking day, ≥ 2 h versus < 2 h	0.0190	1.019	0.691	1.503	0.9235
Nighttime sleep disturbances (severity level, moderate/severe versus mild)	0.0167	1.017	0.744	1.389	0.9166
Troublesome dyskinesia (hours/waking day, ≥ 2 h versus < 2 h)	0.3610	1.435	0.827	2.490	0.1993
Non-motor fluctuations present (yes versus no)	0.1763	1.193	0.893	1.593	0.2322
“Off” time at least every 3 h (yes versus no)	0.3944	1.483	0.978	2.251	0.0638
≥ 5 times daily oral levodopa dosing (yes versus no)	0.7417	2.100	1.544	2.854	**< 0.0001** ^a^
Activities of daily living limitation (severity level, moderate/severe versus mild)	0.5389	1.714	1.213	2.422	**0.0022** ^a^
Falling (frequency, most/all the time versus some of the time)	0.0432	1.044	0.397	2.744	0.9302
Dementia (severity level, moderate/severe versus mild)	−0.4134	0.661	0.399	1.097	0.1093
Psychosis (severity level, moderate/severe versus mild)	0.5745	1.776	0.846	3.731	0.1291

^a^Correlations with physician’s judgment were significant for the Delphi criteria ≥5 times daily oral levodopa dosing (*p* < 0.0001) and activities of daily living limitation (*p* = 0.0022). *APD* advanced Parkinson’s disease, *CI* confidence interval

### DAT eligibility according to physician’s judgment

Of 1342 APD patients, 882 (66%) patients were eligible for DAT and 34% (*n* = 460) were ineligible. Of 1272 non-APD patients, 127 (10%) patients were eligible for DAT. Of 882 APD DAT-eligible patients, 384 (43.6%) had ongoing DAT, 164 (18.6%) had decided to initiate DAT during the study visit, and 332 (37.7%) had no DAT planned (data were unavailable for two subjects). Patients deemed eligible but who had no DAT planned listed the need for more time to decide (39%) and/or patient refusal (25%) as primary reasons for not using DAT (Table 5).

**Table 5:** Reasons patients with APD were not using DAT

Reason^a^	Patients, n (%), *n* = 332
Patient characteristic reasons	
Cognitive-related issues	27 (8)
Psychiatric-related issues	23 (7)
Comorbidities	20 (6)
Age	12 (4)
Lack of caregiver/family support	11 (3)
Motor function–related issues	6 (2)
Reasons related to patient decision	
Patient needs more time to decide	129 (39)
Patient refusal	82 (25)
Other reasons	
Cost/reimbursement	0
Other reasons	45 (14)

^a^Multiple entries for each patient were possible. A total of 23 patients who gave reasons related to patient decisions and had physician-based patient characteristic reasons were excluded

*APD* advanced Parkinson’s disease, *DAT* device-aided treatment

A comparison of baseline characteristics by DAT eligibility is shown in Table 6. Of 384 patients currently on DAT, 57% were using DBS, 39% were using LCIG, and 8% were receiving CSAI (patients were not limited to only one DAT). Mean (SD) duration of DBS treatment, LCIG therapy, and CSAI was 39.9 (41.5) months, 20.3 (20.1) months, and 22.6 (27.6) months, respectively. The number of current PD treatments per APD patient ranged from one to six (one, 19.7%; two, 33.6%; three, 28.5%; four, 12.0%; five, 3.8%; and six, 0.3%).

**Table 6:** Baseline characteristics of DAT-eligible and DAT-ineligible patients with APD

Characteristics	DAT-eligible, *n* = 882^a^			
	Ongoing DAT *n* = 384	Planned DAT *n* = 164	Not planned *n* = 332	DAT-ineligible *n* = 460
Demographics				
Age, years, mean (SD)	65.1 (8.7) *n* = 383	64.3 (9.0) *n* = 163	67.8 (9.6) *n* = 330	70.6 (9.1)
Sex, male, n (%)	239 (62)	103 (63)	200 (60)	274 (60)
Caregiver support, n (%)	278 (73) *n* = 380	109 (67) *n* = 162	242 (74) *n* = 328	286 (63)
Medical history				
PD duration, years, mean (SD)	14.2 (5.6) *n* = 372	10.1 (4.7) *n* = 160	11.1 (5.0 *n* = 322	8.6 (5.5) *n* = 449
Motor fluctuations, n (%)	353 (92)	154 (94)	320 (96)	338 (74)
Motor fluctuation duration years, mean (SD)	7.2 (4.3) *n* = 351	4.2 (2.8) *n* = 152	4.4 (3.5) *n* = 312	3.3 (2.9) *n* = 334
Comorbidity, n (%)	327 (85)	135 (82)	301 (91)	431 (94)
Time since referral to center, years, mean (SD)	5.2 (5.5) *n* = 305	2.7 (3.7) *n* = 119	4.7 (4.8) *n* = 231	4.0 (4.6) *n* = 340

^a^two patients had missing data

*APD* advanced Parkinson’s disease, *DAT* device-aided treatment, *PD* Parkinson’s disease, *SD* standard deviation

**Fig. 2:**
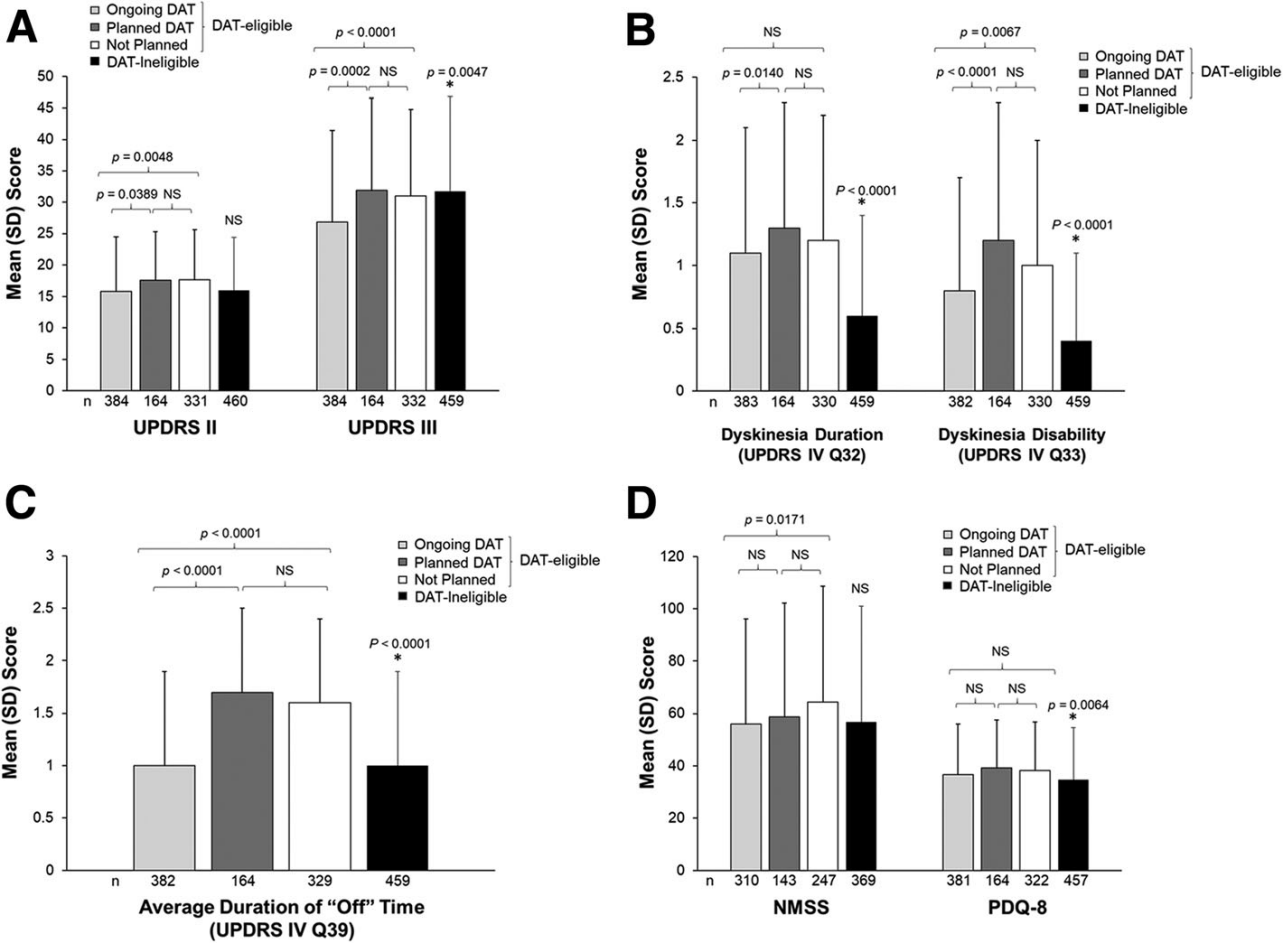
Clinical PD assessments based on DAT eligibility and status. Comparisons between DAT groups (ongoing, planned, not planned, and ineligible) are shown for (**a**) activities of daily living (UPDRS II) and motor symptom severity (UPDRS III), **b** dyskinesia duration and disability (UPDRS Part IV Q32 and Q33), **c** average duration of “Off” time (UPDRS Part IV Q39), and (**d**) NMS burden and QoL (PDQ-8). Error bars indicate the standard deviation. *p* values from a paired *t* test indicate statistical significance. An asterisk indicates significant differences between DAT-ineligible patients and all DAT-eligible patients combined. DAT = Device-aided treatment; NMSS = Non-motor Symptom Scale; NS = Not significant; PDQ-8 = Parkinson’s disease 8-item questionnaire; SD = Standard deviation; UPDRS = Unified Parkinson’s Disease Rating Scale

Patients with APD currently receiving DAT had significantly lower impairment in ADL compared with APD patients who had planned DAT (*p* = 0.0389) and compared with APD patients who had no DAT planned (*p* = 0.0048) (Fig. 2a). Patients who were currently receiving DAT had significantly lower impairment in motor symptoms compared with patients who had planned DAT (*p* = 0.0002) and those who had no DAT planned (*p* < 0.0001); motor symptom severity was also significantly lower in the combined DAT-eligible group compared with DAT-ineligible patients (*p* = 0.0047).

Patients currently receiving DAT had significantly lower scores for dyskinesia duration than those with planned DAT (*p* = 0.0140). Dyskinesia disability was significantly lower for patients with ongoing DAT compared with patients who had DAT planned (*p* < 0.0001) and with patients who had no DAT planned (*p* = 0.0067) (Fig. 2b). Average duration of “Off” time was significantly lower for patients receiving ongoing DAT compared with patients who had planned DAT (*p* < 0.0001), and with patients who had no DAT planned (*p* < 0.0001) (Fig. 2c). Patients currently receiving DAT had significantly lower NMSS total scores than patients with no DAT planned (*p* = 0.0171) (Fig. 2d).

Dyskinesia duration and disability scores and “Off” time were significantly lower in DAT-ineligible patients than in DAT-eligible patients who are not yet treated (*p* < 0.0001 for all). QoL was significantly better in DAT-ineligible patients than in the combined DAT-eligible patient group (*p* = 0.0064).

## Discussion

The progressive nature of PD means that categorical sorting of patients into distinct disease stages might help to ensure implementation of best practices and effective treatment strategies. As PD progresses, oral pharmacotherapeutic strategies may not be as efficient in some patients, and more invasive DAT (e.g. continuous-infusion therapies [CSAI and LCIG] and DBS) need to be initiated to manage symptoms and maintain QoL [5]. Clinical indicators for categorizing APD are ambiguous and subjective, presenting unmet needs for enhanced descriptions and guidelines relating to the staging of APD and precise timeframes for DAT implementation. Current classification methods based on motor symptom trajectory do not provide a comprehensive patient profile to accurately characterize the multidimensional features of motor and non-motor manifestations accompanying APD. The current study aimed to determine the proportion of PD patients identified as having APD according to physician’s assessment, to compare the clinical features of APD and non-APD patients, and to compare newly developed Delphi criteria for APD with physician’s judgment. The current study is the first report to include international data on the proportion of APD, clinical features, evaluation of physician’s judgment, use of the Delphi criteria in routine clinical practice, gaps in patient identification, and standard patient scores.

The key limitations of the regularly used Hoehn & Yahr staging is the neglect of non-motor symptoms and levodopa-induced complications (e.g. motor fluctuations, dyskinesia) which strongly contribute to impaired QoL in APD [11]. We found a moderate correlation between physician’s judgment and Delphi criteria for APD, indicating some consensus already. Approximately half of the PD patients at movement disorder clinics were classified by the physician as APD, although the range varied considerably in certain countries. These variations highlight the inconsistencies among health systems, movement disorders clinics, and physicians in differing regions to categorize APD and may be based on different intercultural disease perceptions and treatment availabilities. The high proportion of APD classification (i.e. 51%) by clinician judgment is not meant to represent an epidemiological prevalence in the PD patient population as judgment is likely biased by patient selection from only movement disorder centers that tend to treat more advanced-stage PD patients. Patients who were characterized as having APD had worse motor and NMS severity and reduced QoL compared with non-APD patients. This is consistent with findings from other studies showing disease characteristics [12] and motor and non-motor features in APD [13]. Both groups were similar regarding age, sex, and living situation, but differed in terms of caregiver support status, which is likely an effect of having APD.

Delphi criteria have been previously used by other investigators to define APD. The CEPA study [14] categorized APD symptoms by definitive symptoms, probable symptoms, and possible symptoms. The authors’ list of symptom coverage is extensive and generally covered by our targeted Delphi criteria, which allows a more structured and simplistic method of assessment. Longer time since diagnosis, dyskinesia duration, and limitations of ADL are reported to be predictors for the diagnosis of APD [6]. That 87.0% of APD (versus 23.2% in non-APD) patients were reported as having motor fluctuations may hint that physician’s judgment was strongly based on motor fluctuations. Compared with Delphi criteria for APD, which subsumes a broader range of symptoms under the diagnosis of APD, physician’s judgment, based primarily on motor fluctuations, is too narrow.

Of APD patients, 66% were considered eligible for DAT. Within this group, many patients had ongoing DAT or had made the decision to initiate DAT. However, almost one-third of DAT-eligible patients did not plan on initiating DAT at the time of their study visit, demonstrating that patients did not elect to follow their clinician’s suggested optimal treatment plan. Most DAT-eligible patients were not currently using DAT based on personal decision (outright refusal or the need for more time to decide). Transitioning from oral drug administration to more invasive measures is an important step for patients; therefore, it is not surprising that many patients initially decline to move forward with DAT or request more time to discuss and educate themselves prior to initiating the change in treatment. The large percentage of patients delaying their DAT initiation suggests a need for enhanced patient-physician guidance on navigating the transition to DAT for optimal patient outcomes, while still incorporating patient preferences [15, 16].

Further evaluations compared patient clinical outcomes as they related to DAT eligibility status. Generally, DAT-eligible patients had worse motor symptoms and QoL than did DAT-ineligible patients. Patients who were currently receiving DAT at the time of the study had significantly lower impairment in ADL, motor function, dyskinesia duration and disability, “Off” time, and lower NMSS total scores than did patients who were not currently on DAT (whether or not they were planning to initiate DAT). These findings are similar to other studies that demonstrated DAT improvement of motor and non-motor complications associated with APD [17, 18]. The fact that the Delphi criteria include two items on NMS (nighttime sleep and non-motor fluctuations) may make it more suitable to obtain a comprehensive patient profile to determine advanced PD or DAT eligibility. Evidence from the GLORIA registry, which sampled the largest cohort of advanced PD patients treated with LCIG in routine clinical care, showed NMS improvements in patients treated with DAT (i.e. LCIG) in multiple NMSS domains [19, 20]. These findings further demonstrate that non-motor features of APD can be improved with optimized dopaminergic delivery. Of note, not all APD patients are candidates for DAT (eg, patients with dementia) and some non-APD patients may still be candidates for DAT because they have demanding functional needs (eg, young working individuals), they may be using DAT for the (non-approved) treatment of non-motor symptoms or for treatment of PD-related tremor, or they may have poor tolerability of oral levodopa. However, the reasons for non-APD use of DAT were not captured in the study and therefore remain speculative.

Patient baseline characteristics are in keeping with clinical reports in which DATs are implemented. Weaver and colleagues [18] report patient characteristics for 121 dB candidates with APD, showing similar age and living situation but fewer patients with caregiver support compared with patients in the current study. Patients in the current study who were receiving DBS have similar scores as noted in the DAT-eligible group in UPDRS II assessments but scored lower in motor function as determined by UPDRS III compared with that reported by Weaver et al. [18]. A report from Garcia Ruiz and colleagues [17] summarizes patient characteristics from 20 open-label studies using CSAI in patients with APD. DAT-eligible patients from the current study showed similar duration of disease but lower mean age compared with all CSAI studies. Patients treated with LCIG also demonstrated similar age of DAT onset, duration of disease, and gender ratio [21].

This study utilized practicing clinicians and a large sample size of patients from multiple countries who were currently seeking medical support. It is important to note that the study was not designed to be an epidemiological study and the goal was not specifically to determine the percentage of APD patients (prevalence) using that methodology. The collected data demonstrate real-world evidence of treatment patterns and highlight gaps in current clinical care and possible areas for improvement. A study limitation and bias were noted regarding recruitment of inpatients at movement disorder clinics, possibly resulting in increased prevalence of (hospitalized) patients with more advanced PD. Clinical sites were chosen based on, among other aspects, availability of DAT. This likely contributed to the disproportionate representation of DAT modalities (i.e. more patients using LCIG). A bias may have occurred when the investigators were trained for the study where the investigators were informed about the Delphi criteria, set up by their peer movement disorder specialists, which may potentially have influenced their understanding and judgment. Another limitation to the study is the post hoc nature of the analysis regarding DAT eligibility. In addition, study results were only obtained from a single, independent patient visit. Without follow-up, it is unknown if DAT-eligible patients who were not currently using DAT have changed their minds and chose DAT or if DAT-ineligible patients have had a change in their DAT eligibility after this study concluded.

The present study reports findings from a cross-sectional, international, observational study of patients with PD. This study highlights the inconsistency in physician judgment for DAT eligibility and the actual number of patients considering it. The observed heterogeneity in patient identification results in increased numbers of patients with a delayed diagnosis of APD and, therefore, higher numbers of patients who do not receive optimized medical treatment.

## Conclusions

There is a need for consistent criteria by which APD can be detected earlier, thereby allowing patients to adequately prepare for changing medical needs that arise in advancing stages of the disease. The Delphi criteria highlight some key criteria that are both quantifiable and tangible for the patient and the physician. These criteria will help to increase the awareness for these symptoms and further emphasize the need to screen these symptoms. Improving treatment for this patient population begins with the optimization of oral dopamine replacement therapies, which is dependent on the correct diagnosis of the disease stage and medication requirement. Altogether, these findings aid in further categorization of patients into APD and suggest the Delphi criteria for APD may be a useful assessment tool to aid patient classification and demonstrate the benefit of DAT in APD patients. Further studies are needed to validate and evaluate the use of these criteria in routine clinical practice.

## Additional file

Additional file 1 **Table S1.** Types of current Parkinson’s disease treatment. **Table S2.** Sensitivity analysis of correlations between physician’s judgment of APD and individual Delphi criteria for APD (ongoing DAT patients excluded). **Table S3.** Association between physician’s judgment of APD and individual prognostic parameters. (PDF 57 kb)

## Abbreviations

ADL: Activities of daily living
APD: Advanced Parkinson’s disease
CSAI: Continuous subcutaneous apomorphine infusion
DBS: Deep brain stimulation
LCIG: Levodopa-carbidopa intestinal gel
NMS: Non-motor symptoms
NMSS: Non-motor Symptom Scale
PD: Parkinson’s disease
PDQ-8: 8-item Parkinson’s Disease Quality of Life Questionnaire
QoL: Quality of life
UPDRS: Unified Parkinson’s Disease Rating Scale
