# Supplementary material for: Characterizing advanced Parkinson’s disease: OBSERVE-PD observational study results of 2615 patients
Source: BMC Neurol. 2019 Apr 2;19:50. doi: 10.1186/s12883-019-1276-8 (PMC6444751; doi:10.1186/s12883-019-1276-8)
Supplement: Supplementary file 1 — Table S1. Types of current Parkinson’s disease treatment. Table S2. Sensitivity analysis of correlations between physician’s judgment of APD and individual Delphi criteria for APD (ongoing DAT patients excluded). Table S3. Association between physician’s judgment of APD and individual prognostic parameters. (PDF 57 kb) [file 12883_2019_1276_MOESM1_ESM.pdf]

Table S1. Types of current Parkinson's disease treatment

	n (%)		
	APD according to physician's judgment		
Type of PD medication	APD (n = 1342)	Non-APD (n = 1273)	Total (n = 2615)
Oral levodopa/carbidopa or benserazide	1227 (91%)	983 (77)	2210 (85)
Oral dopamine agonist(s)	699 (52)	600 (47)	1299 (50)
Apomorphine patch	14 (1)	6 (0.5)	20 (0.8)
Apomorphine s.c. rescue injection	29 (2)	1 (0.1)	30 (1)
COMT inhibitors	411 (31)	133 (10)	544 (21)
MAO-B inhibitors	336 (25)	421 (33)	757 (29)
Amantadine	334 (25)	158 (12)	492 (19)
Other	185 (14)	137 (11)	322 (12)
Missing	29 (2)	52 (4)	81 (3)

COMT catechol-o-methyltransferase, *MAO-B* monoamine oxidase B, *PD* Parkinson's disease, *s.c.* subcutaneous.

Table S2 Sensitivity analysis of correlations between physician's judgment of APD and individual Delphi criteria for APD (ongoing DAT patients excluded)

	Odds ratio		
Prognostic parameter	Cohen's Kappa estimate	95% CI	
Overall APD classification by Delphi method	0.430	0.397	0.464
Troublesome motor fluctuations (severity level, moderate/severe versus mild)	0.502	0.467	0.537
"Off" time (hours/waking day, ≥ 2 hours versus < 2 hours)	0.461	0.424	0.498
Nighttime sleep disturbances (severity level, moderate/severe versus mild)	0.262	0.223	0.302
Troublesome dyskinesia (hours/waking day, ≥ 2 hours versus < 2 hours)	0.283	0.250	0.316
Non-motor fluctuations present (yes versus no)	0.363	0.323	0.402
"Off" time at least every 3 hours (yes versus no)	0.383	0.347	0.418
≥ 5 times daily oral levodopa dosing (yes versus no)	0.497	0.461	0.533
Activities of daily living limitation (severity level, moderate/severe versus mild)	0.473	0.436	0.510
Falling (frequency, most/all the time versus some of the time)	0.098	0.077	0.119
Dementia (severity level, moderate/severe versus mild)	0.106	0.077	0.134
Psychosis (severity level, moderate/severe versus mild)	0.062	0.043	0.082

APD advanced Parkinson's disease, *CI* confidence interval, *DAT* device-aided treatment

Table S3 Association between physician's judgment of APD and individual prognostic parameters

Prognostic parameter	Regression coefficient	Adjusted estimate	Odds ratio 95% Wald CI		*p* value
Age	0.00266	1.003	0.988	1.018	0.7262
UPDRS II score	0.0365	1.037	1.003	1.073	0.0329
UPDRS III score	–0.00395	0.996	0.981	1.011	0.6110
UPDRS V score	0.8229	2.277	1.744	2.973	<0.0001
UPDRS IV item 32 (26%–100% of the day versus 0%–25% of the day)	0.1413	1.152	0.645	2.056	0.6326
UPDRS IV item 33 (moderately/severely/completely disabling versus not/mildly disabling)	–0.3688	0.692	0.368	1.299	0.2515
UPDRS IV item 34 (moderate/severe/marked versus slight/no painful dyskinesia)	0.4532	1.573	0.729	3.393	0.2478
UPDRS IV item 39 (26%–100% of the day versus 0%–25% of the day)	0.1807	1.198	0.782	1.836	0.4069
NMSS score	–0.00481	0.995	0.990	1.000	0.0468
PDQ-8 summary index	0.00636	1.006	0.996	1.016	0.2107
Time since diagnosis	0.1136	1.120	1.085	1.156	<0.0001
Current DAT (yes versus no)	2.0339	7.644	4.830	12.097	<0.0001
Gender (male versus female)	0.3042	1.356	1.035	1.775	0.0270
Race (non-white versus white)	–0.6549	0.519	0.178	1.516	0.2307
Education (less than high school versus high school or higher)	0.0856	1.089	0.794	1.494	0.5955
Motor fluctuations (yes versus no)	1.4507	4.266	3.151	5.777	<0.0001
Caregiver support (yes versus no/not applicable)	0.5016	1.651	1.227	2.223	0.0009
Comorbidities (yes versus no)	0.0181	1.018	0.679	1.528	0.9304
Geographic region (Eastern Europe/ Japan/Asia Pacific versus Western Europe)	0.3450	1.412	1.063	1.876	0.0172

Occupation (unemployed/on sick-leave/retired/home maker/other versus student/[self-]employed)	0.0330	1.034	0.704	1.517	0.8660
Type of residence (at a nursing home/other versus at home)	1.1583	3.184	1.204	8.422	0.0196

APD advanced Parkinson's disease, *CI* confidence interval, *NMSS* Nonmotor Symptom Scale, *PD* Parkinson's disease, *PDQ-8* 8-item Parkinson's Disease Questionnaire, *UPDRS* Unified Parkinson's Disease Rating Scale
